# Influence of pre-endoscopic duration of esophageal food impaction on endoscopy time and postprocedure adverse events

**DOI:** 10.3389/fgstr.2022.935447

**Published:** 2022-08-15

**Authors:** Daniel Anthony DiLeo, Cameron Zenger, Giulio Quarta

**Affiliations:** Department of Medicine, New York University (NYU) Grossman School of Medicine, New York, NY, United States

**Keywords:** food impaction, eosinophilic esophagitis, dysphagia, esophagus, foreign body

## Abstract

**Objectives:**

Esophageal food bolus impaction is a medical emergency and 10-20% of impacted food boluses will require endoscopic removal. Emergent and prolonged procedures are associated with increased adverse events. We are interested in the relationship between the pre-endoscopic duration of esophageal food impaction and the duration of esophagogastroduodenoscopy (EGD) performed to remove the impacted food bolus.

**Methods:**

Between 2010 and 2021, we examined EGD procedures performed for esophageal food impaction. Subjects were classified according to pre-endoscopic duration of food impaction.

**Results:**

We found a positive correlation between pre-endoscopic duration of food impaction and procedure length (r=0.18). Esophageal impactions with mixed foods resulted in the longest procedure duration (p<0.05). Increasing age, male gender, and duration of impaction greater than 42 hours were significantly associated with increased procedure duration (p<0.05). Esophageal perforations, prolonged intubations, admissions following EGD, and readmissions were associated with EGD duration greater than 25.5 minutes. No adverse events occurred in patients who underwent EGD within 6 hours of symptom onset.

**Conclusions:**

In the case of an esophageal food impaction, the time between symptom onset and endoscopy is positively correlated with procedure length and risk of adverse outcomes. We suggest that food impaction should remain an indication for emergent endoscopy. Prospective studies evaluating the safety and outcomes of prolonged time to endoscopy will further clarify the management of esophageal food impactions.

## Introduction

Esophageal food bolus impaction is a medical emergency with an increasing incidence over time (1). Food, specifically meat, is the most common esophageal foreign body in adults (2, 3). Esophageal adverse events of food impactions include ulcers, lacerations, erosions, and perforation (4). Extraesophageal adverse events include aspiration pneumonia, arrhythmias, and hypotension requiring vasopressors (5). While 80% to 90% of impacted food boluses will pass spontaneously, 10% to 20% of impacted food boluses will require endoscopic removal (2).

Endoscopic treatments for esophageal food impactions include advancement of the food bolus into the stomach with pressure from the gastroscope tip and extraction of the food bolus using grasping or suction devices (3). Glucagon is commonly used as an initial attempt to medically manage food impactions, though studies have failed to demonstrate a clear benefit (6). Esophagogastroduodenoscopy (EGD) should not be delayed for the purpose of a medication trial (1). The American Society for Gastrointestinal Endoscopy recommends removal of an impacted esophageal food bolus within 24 hours, as delay decreases the likelihood of successful removal and increases the risk of adverse events (3).

While delayed EGD for esophageal food impaction can increase adverse events, recent studies have highlighted other factors that increase procedural risk. The largest prospective study evaluating sedation-associated adverse events in gastrointestinal endoscopy found that adverse events of endoscopic procedures were significantly increased in emergent procedures as well as prolonged procedures (7). Therefore, understanding factors that contribute to a prolonged procedure may allow us to modify our sedation choice and endoscopic technique to reduce procedure-related adverse events.

In our practice, the duration of EGD performed for esophageal food impaction varies significantly with procedure times, ranging from minutes to hours. We are interested in the relationship between the pre-endoscopic duration of esophageal food impaction and the duration of EGD performed to remove the impacted food bolus. In our retrospective study, our primary objective is to examine whether the duration of food impaction correlates with the amount of time required to successfully remove the impacted food bolus. Our secondary objectives include examining whether there is a relationship between the underlying esophageal pathology and EGD duration, the type of food ingested and EGD duration, the instrument responsible for the removal of the food bolus and EGD duration, and the number of instruments used to remove the impacted food bolus and EGD duration.

## Methods

We queried our institution’s electronic record for endoscopic procedure documentation (ProVation MD, Wolters Kluwer) to identify patients who underwent EGD for an esophageal food impaction. Search fields paired with our corresponding search terms included “procedure: upper GI endoscopy,” “location: esophagus,” and “findings: food.” We included procedures performed at NYU Langone Medical Center - Tisch Hospital and NYU Langone Medical Center - Brooklyn Hospital in our retrospective chart review. Our electronic medical record (Epic) was used to extract data from the charts of the included patients.

Our definition of esophageal food impaction, adapted from Lenz et al. in 2019, was “a sensation of food being lodged in the throat with associated esophageal obstruction (inability to swallow saliva or water) for a long enough time to present to the emergency room.” (8) We included patients who underwent EGD at our clinical sites between 1/1/2010 and 1/10/2021 for an esophageal food impaction. Subjects were not included in our study if an EGD was not required for removal of an impacted food bolus. Examples of such clinical scenarios include spontaneous passage of an impacted food bolus or successful medication trial (i.e., glucagon). Subjects were not included if no esophageal food impaction was identified at the time of EGD. Subjects with an incidental finding of food within the esophagus during an EGD performed for an indication other than an esophageal food impaction were not included in the study. We also did not include patients under the age of 18, pregnant women, and prisoners.

**Figure 1:**
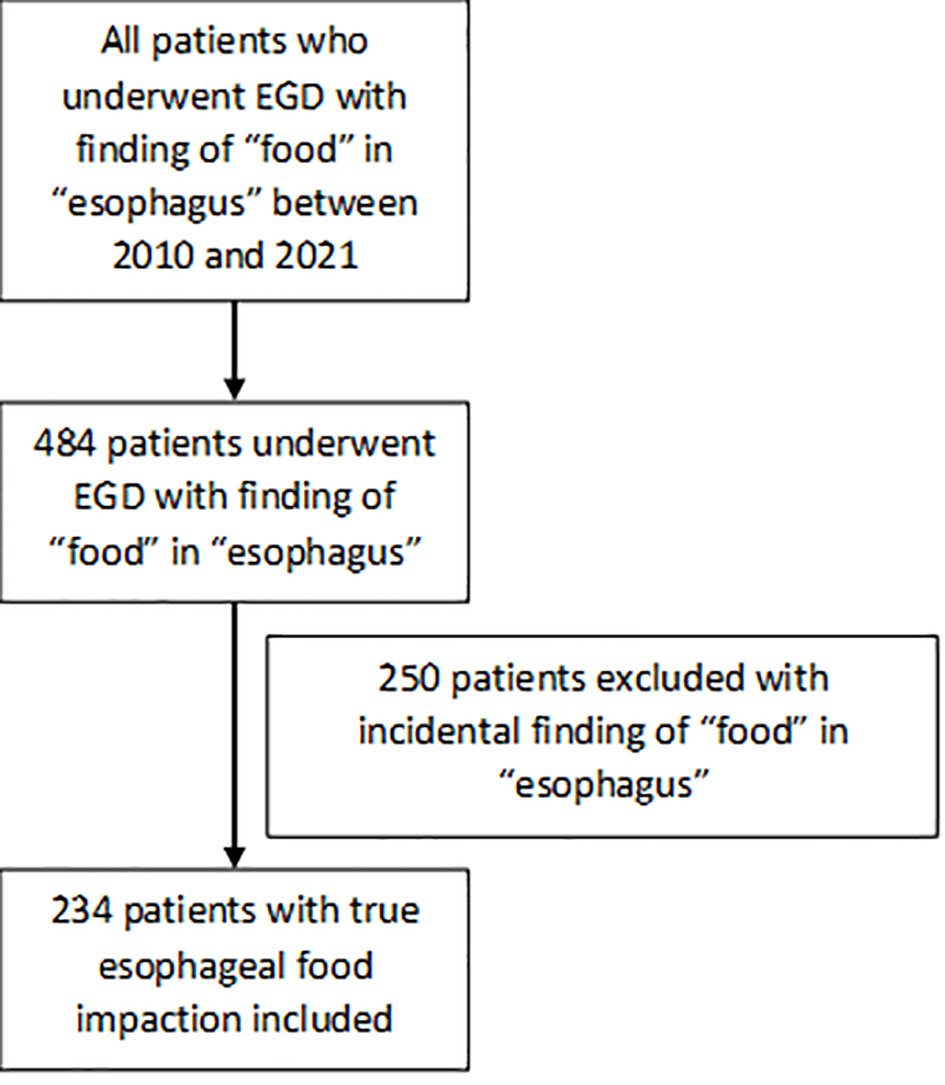
Patient selection flowchart.

A total of 234 EGD procedures performed for esophageal food impaction at our institution between January 1, 2010 to January 1, 2021 were included in our study as shown in **Figure 1**. All procedures were performed by attending physicians and fellows at various levels of training. For each procedure included in our study we recorded the variables outlined in **Supplementary Table 1** (See Table, Supplemental Digital Content 1, which demonstrates the demographic characteristics of the subjects and corresponding procedures).

A number of underlying etiologies have been shown to predispose a patient to an esophageal food impaction including eosinophilic esophagitis, reflux esophagitis, peptic stricture, Schatzki’s ring, motility disorders including achalasia and presbyesophagus, and malignancy (9). In our study, identification of the esophageal pathology responsible for food impaction was based on the impression documented by the gastroenterologist in the EGD procedure note. In cases of suspected eosinophilic esophagitis, we reviewed the pathology results of esophageal biopsies, if available, to ensure the pathology was consistent with eosinophilic esophagitis (i.e., greater than or equal to 15 eosinophils per high power field in proximal and distal esophageal biopsies).

The time of symptom onset was obtained from the initial gastroenterology consultation note. If the time was not specified in the gastroenterology note, the time was obtained from emergency room documentation. Duration of food impaction was divided into five classes, which are listed in **Table 1**. We utilized procedure classes to provide a clinically intuitive way to categorize duration of food impaction and to account for error in reporting the initial time of food impaction.

**Table 1:** Procedure classes by duration of food impaction.

Procedure class	Duration
Class 1	Less than or equal to 6 hours
Class 2	Greater than 6 hours, but less than or equal to 12 hours
Class 3	Greater than 12 hours, but less than or equal to 24 hours
Class 4	Greater than 24 hours, but less than or equal to 48 hours
Class 5	Greater than 48 hours

### Statistical analysis and ethics

Approval was obtained from our institutional review board (project ID: i20-01985). The date of IRB registration was February 1, 2021. The need for individual informed consent was waived for this retrospective analysis of data, with no breach of privacy or anonymity. Thus, data will not be publicly available. Statistical analysis was performed in R (version 3.6.0).

## Results

**Figure 2:**
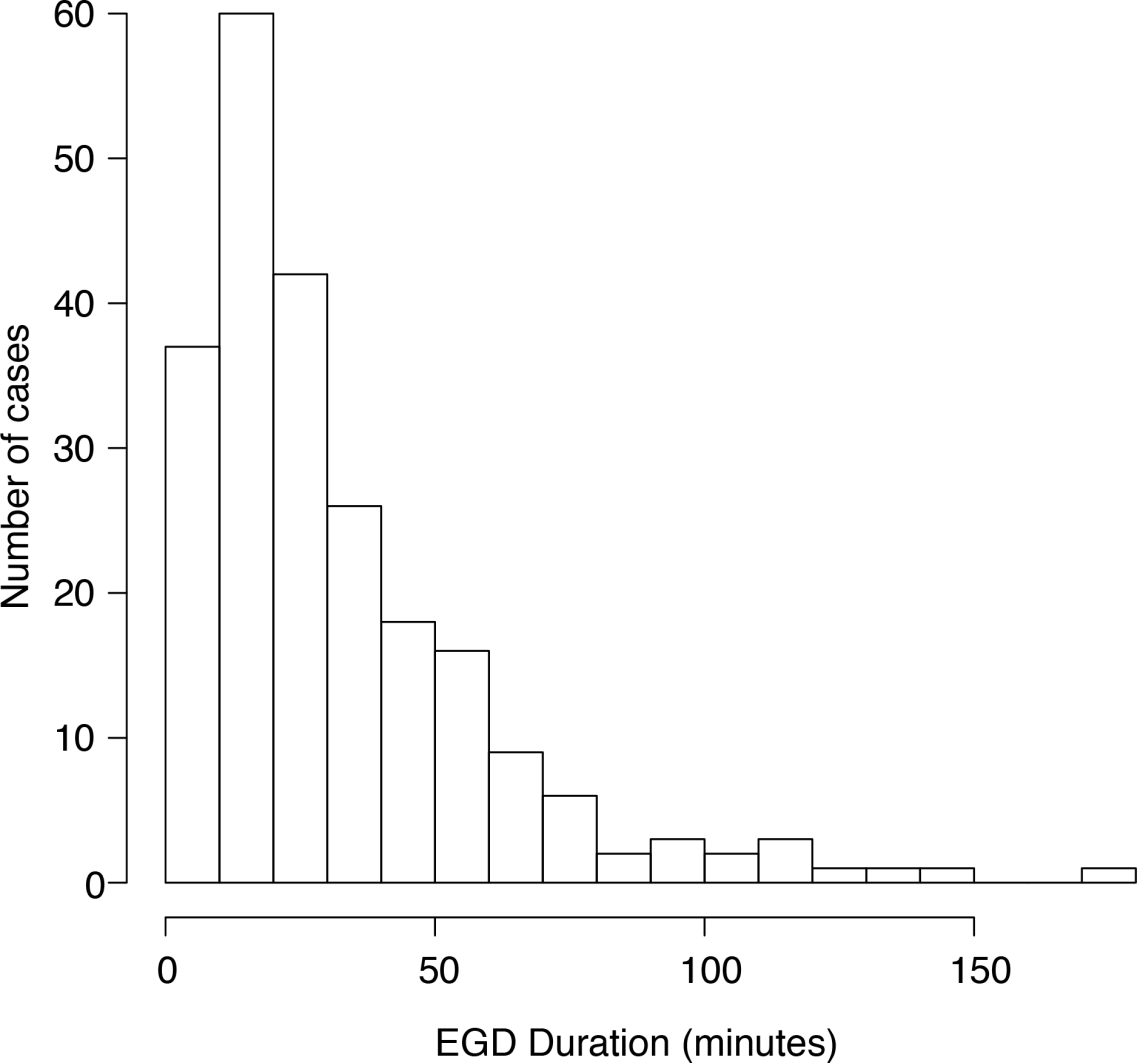
Distribution of esophagogastrodudoenoscopy duration.

Over the time studied, 234 participants met inclusion. The majority of participants were male, with first-time presentations of esophageal food impaction. The median age was 48. Most participants had an American Society of Anesthesiologists (ASA) physician classification of 2, and the overwhelming majority of procedures were performed under general anesthesia. Most food impactions were associated with beef or chicken consumption prior to presentation. EGD procedure duration ranged from 2 minutes to 180 minutes (**Figure 2**). The median EGD duration was 25.5 minutes.

**Figure 3:**
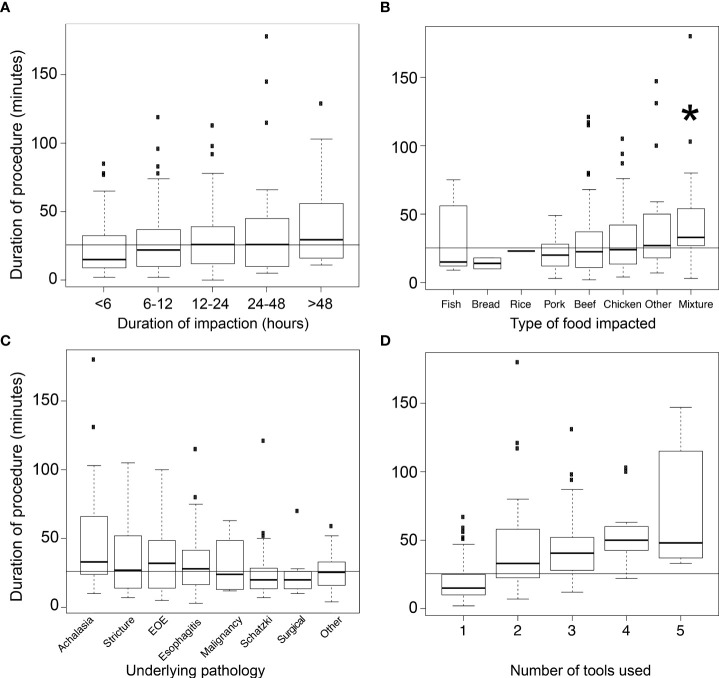
**(A)** Duration of esophagogastrodudoenoscopy for a given procedure class (i.e. duration of food impaction in hours). **(B)** Duration of esophagogastrodudoenoscopy for a given type of food impacted. **(C)** Duration of esophagogastrodudoenoscopy for a given esophageal pathology. **(D)** Duration of esophagogastrodudoenoscopy compared with number of tools needed to sucessfully treat the food impaction. *p < 0.05.

We sought to identify historical factors which influence the duration of EGD performed for food impaction. We found a positive correlation between pre-endoscopic duration of food impaction and procedure length (r=0.18; **Figure 3A**). Esophageal impactions with mixed foods resulted in the longest procedure duration (33 minutes; p<0.05; **Figure 3B**). Esophageal impactions with ingested pork resulted in the shortest procedure duration of 20 minutes (p<0.0001). We found no significant difference between the type of food ingested and the number of instruments used to clear the food bolus.

We examined the relationship between the esophageal pathology contributing to food impaction and the duration of EGD (**Figure 3C**). Achalasia was associated with longer procedure duration (33 minutes), though this difference did not reach statistical significance (p=0.07). Peptic stricture and eosinophilic esophagitis also trended towards longer median procedure durations of 27 and 32 minutes, respectively.

**Figure 4:**
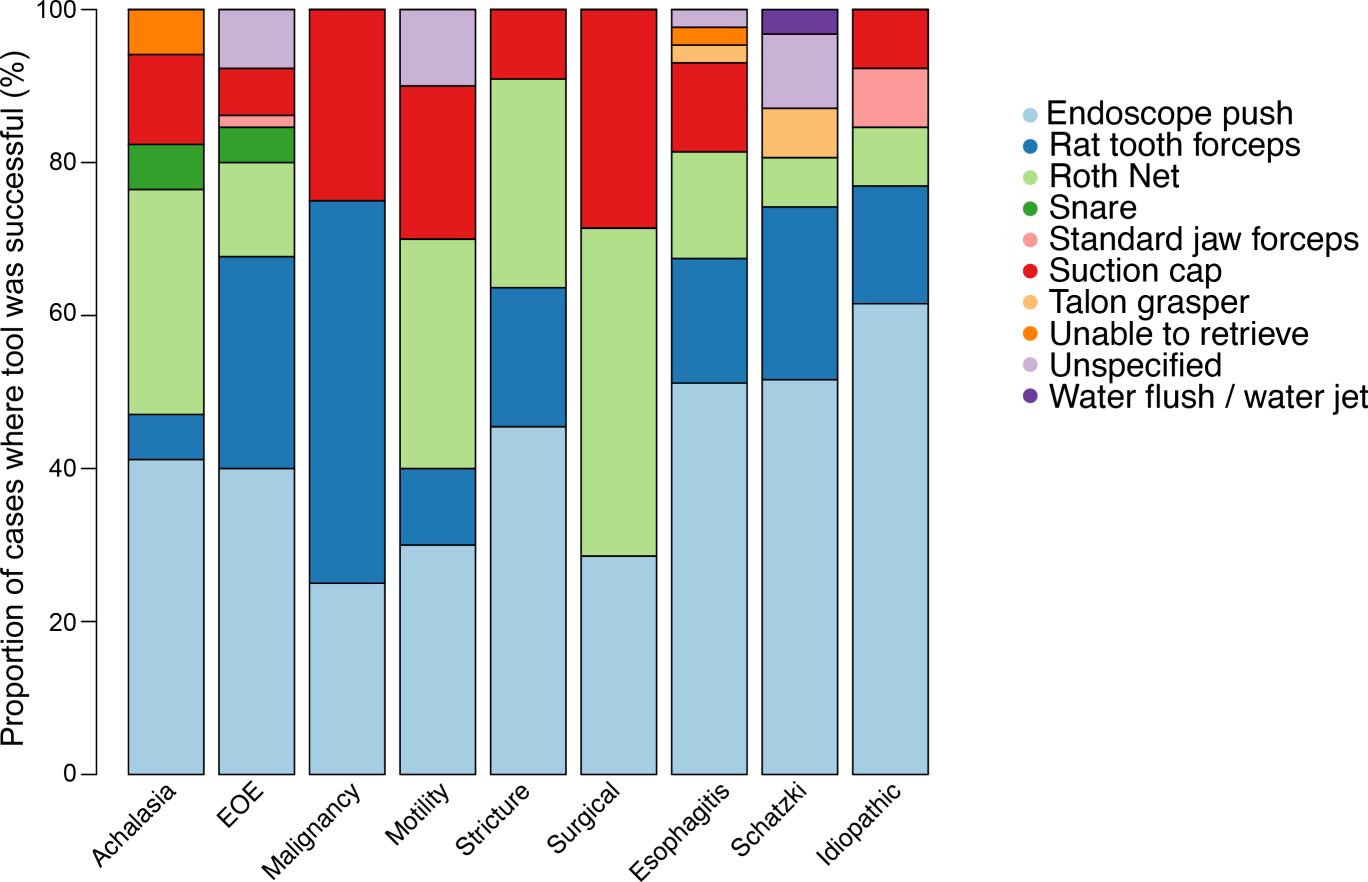
Distribution of instruments responsible for successful removal of impacted food bolus for a given esophageal pathology.

We found a strong positive correlation between the number of instruments used and procedure length (r=0.52; **Figure 3D**). Endoscope push was successful in most cases. In cases where malignancy was responsible, rat toothed forceps were predominantly used to clear the food bolus. In post-surgical etiologies, use of a Roth Net^®^ (STERIS Healthcare, Mentor, OH) was successful in clearance of the food bolus (**Figure 4**).

To develop a model of pre-endoscopic historical factors which influence the duration of endoscopy, we divided cases into those shorter and longer than 25.5 minutes duration. We developed a logistic regression model based on the data from **Table 2**. Increasing age, male gender, and duration of impaction greater than 42 hours were significantly associated with increased procedure duration (p<0.05).

**Table 2:** Procedure characteristics.

Characteristic	Number of subjects
Sex (Male) (%)	144 (61.8)
Age [Median (IQR)]	48 (34 - 70)
Achalasia (%)	13 (5.6)
Eosinophilic esophagitis (%)	47 (20.2)
Prior history of food impaction (%)	98 (42.2)
Overtube used (%)	21 (9.7)
Glucagon used (%)	164 (72.6)
General anesthesia (%)	208 (91.2)
Type of food impacted	
Chicken	56
Beef	70
Pork	22
Bread	2
Rice	1
Fish	9
Mixture	37
Other	22

We hypothesized that prior knowledge of these historical factors may influence the outcome of endoscopic retrieval; thus we sought to determine if there is an association between EGD duration and patient outcomes. Perforation (n=2), prolonged intubation (n=2), and readmission (n=1) only occurred with EGD longer than 25.5 minutes. Admission after EGD (n=22) occurred more frequently if duration was prolonged beyond 25.5 minutes (3.6% vs. 18.8%; p = 0.0017). There were no adverse events that occurred in procedure class 1, i.e., in patients who underwent EGD within 6 hours of symptom onset. We conclude that prolonged EGD for food impaction is associated with worse patient outcomes.

## Discussion

Esophageal food impaction is a medical emergency and prompt removal of the impacted food bolus is recommended to avoid esophageal and extraesophageal adverse events (3). We found that in the case of an esophageal food impaction, the time between symptom onset and endoscopy is positively correlated with procedure length and risk of adverse outcomes. Adverse events were associated with EGD duration greater than 25.5 minutes, while no adverse events in our study occurred in patients who underwent EGD within 6 hours of symptom onset. Prolonged endoscopy has been associated with increased aspiration risk (10). Endoscopists should be aware of any factors that might influence EGD duration to identify potentially high-risk patients.

Recent literature demonstrated the rate of adverse events was similar in patients with esophageal food impaction who underwent endoscopy within 12 hours or after 12 hours of symptom onset (11). As our study suggests that increased time to endoscopy is associated with prolonged procedure duration, and that prolonged procedure duration is associated with adverse events, we suggest that food impaction remain an indication for emergent endoscopy until the safety of delayed endoscopy is clarified with prospective studies.

We also investigated whether underlying esophageal pathology leading to food impaction influences EGD duration. We hypothesized endoscopy in patients with achalasia would be prolonged as patients with achalasia are able to tolerate retention of larger amounts of food due to chronic esophageal dilation and diminished esophageal mechanosensitivity and chemosensitivity (12). In our cohort, patients with achalasia had the longest EGD length with a median procedure time of 33 minutes. However, this difference did not reach statistical significance. In our practice we predominantly use general anesthesia when performing EGD in patients with achalasia for the purpose of airway protection given the potential for prolonged procedures, as well as the high likelihood of encountering an esophagus filled with food and debris. Use of an overtube in patients with achalasia-associated food impactions is recommended in anticipation of requiring multiple passages of the gastroscope (13).

We found a strong positive correlation between procedure duration and number of instruments used to remove the impacted food bolus. We hypothesize that a highly fixed food impaction requires the use of multiple instruments and thus extends the time of the procedure. Endoscope push was successful in the majority of cases. This technique has been shown to be safe and effective in managing cases of esophageal food impactions (14, 15). In patients with esophageal food impaction due to malignancy and post-surgical etiology, use of rat toothed forceps and Roth Net^®^ was most successful in clearance of the food bolus, respectively. These findings may help us choose an endoscopic tool for removal of an impacted food bolus for a specific esophageal pathology.

Our study has several limitations. The study is retrospective in design and is at risk of bias. We rely on documentation of time of food impaction by our providers, which is limited by the accuracy of the medical record. We have also identified variables which may influence procedure duration. In cases of eosinophilic esophagitis, some endoscopists may choose to take esophageal biopsies for diagnostic purposes or to assess response to therapy. Esophageal biopsies may prolong the duration of EGD beyond the time needed to remove the impacted food bolus. Operator experience and technique may also influence the duration of esophagogastroduodenoscopy.

Overall, we have demonstrated there is a positive association between pre-endoscopic duration of esophageal food impaction and procedure length. We identified multiple variables that may influence EGD duration and contribute to post-procedure adverse events. Prospective studies evaluating the safety and outcomes of prolonged time to endoscopy will further clarify the management of esophageal food impactions.

## Data availability statement

The original contributions presented in the study are included in the article/**Supplementary Material**. Further inquiries can be directed to the corresponding author.

## Ethics statement

The studies involving human participants were reviewed and approved by Institutional Review Board Operations - NYU Langone Health. The ethics committee waived the requirement of written informed consent for participation.

## Author contributions

DD wrote the manuscript, obtained data, and helped conceive the study design. GQ conceived the study design, performed statistics, created figures, and edited the manuscript. CZ helped obtain data and edited the manuscript. All authors contributed to the article and approved the submitted version.

## Conflict of interest

The authors declare that the research was conducted in the absence of any commercial or financial relationships that could be construed as a potential conflict of interest.

## Publisher’s note

All claims expressed in this article are solely those of the authors and do not necessarily represent those of their affiliated organizations, or those of the publisher, the editors and the reviewers. Any product that may be evaluated in this article, or claim that may be made by its manufacturer, is not guaranteed or endorsed by the publisher.
