# Supplementary material for: Influence of pre-endoscopic duration of esophageal food impaction on endoscopy time and postprocedure adverse events
Source: Front Gastroenterol (Lausanne). 2022 Aug 15;1:935447. doi: 10.3389/fgstr.2022.935447 (PMC12952350; doi:10.3389/fgstr.2022.935447)
Supplement: Supplementary file 1 [file Table_1.docx]

| **Variable** |
| --- |
| Age |
| Gender |
| Past medical history |
| Surgical history |
| History of eosinophilic esophagitis (yes / no) |
| History of achalasia (yes / no) |
| Prior history of esophageal food impaction or foreign body impaction (yes / no) |
| Time between symptom onset and EGD start time (duration of food impaction) |
| Esophagogastroduodenoscopy procedure duration |
| Type of anesthesia used |
| Overtube used (yes / no) |
| Glucagon used (yes / no) |
| Type of food impacted |
| Number of instruments required to successfully remove impacted food bolus |
| Instrument responsible for successful removal of impacted food bolus |
| Procedure complications |
| Esophageal pathology suspected / confirmed to be responsible for food impaction |

**Supplementary Table 1** – Demographic characteristics
